# Pangenome Identification and Analysis of Terpene Synthase Gene Family Members in *Gossypium*

**DOI:** 10.3390/ijms25179677

**Published:** 2024-09-06

**Authors:** Yueqin Song, Shengjie Han, Mengting Wang, Xueqi Ni, Xinzheng Huang, Yongjun Zhang

**Affiliations:** 1 College of Horticulture and Plant Protection, Henan University of Science and Technology, Luoyang 471023, China; songyueqin6@163.com (Y.S.); hanshengjie1029@163.com (S.H.); 2 MOA Key Lab of Pest Monitoring and Green Management, Department of Entomology, College of Plant Protection, China Agricultural University, Beijing 100193, China; wangmt@cau.edu.cn (M.W.); xueqini@163.com (X.N.); 3 State Key Laboratory for Biology of Plant Diseases and Insect Pests, Institute of Plant Protection, Chinese Academy of Agricultural Sciences, Beijing 100193, China

**Keywords:** cotton, *Helicoverpa armigera*, *Apolygus lucorum*, atypical

## Abstract

Terpene synthases (TPSs), key gatekeepers in the biosynthesis of herbivore-induced terpenes, are pivotal in the diversity of terpene chemotypes across and within plant species. Here, we constructed a gene-based pangenome of the *Gossypium* genus by integrating the genomes of 17 diploid and 10 tetraploid species. Within this pangenome, 208 *TPS* syntelog groups (SGs) were identified, comprising 2 core SGs (TPS5 and TPS42) present in all 27 analyzed genomes, 6 softcore SGs (TPS11, TPS12, TPS13, TPS35, TPS37, and TPS47) found in 25 to 26 genomes, 131 dispensable SGs identified in 2 to 24 genomes, and 69 private SGs exclusive to a single genome. The mutational load analysis of these identified *TPS* genes across 216 cotton accessions revealed a great number of splicing variants and complex splicing patterns. The nonsynonymous/synonymous Ka/Ks value for all 52 analyzed *TPS* SGs was less than one, indicating that these genes were subject to purifying selection. Of 208 *TPS* SGs encompassing 1795 genes, 362 genes derived from 102 SGs were identified as atypical and truncated. The structural analysis of *TPS* genes revealed that gene truncation is a major mechanism contributing to the formation of atypical genes. An integrated analysis of three RNA-seq datasets from cotton plants subjected to herbivore infestation highlighted nine upregulated *TPSs*, which included six previously characterized *TPSs* in *G. hirsutum* (*AD1_TPS10*, *AD1_TPS12*, *AD1_TPS40*, *AD1_TPS42*, *AD1_TPS89*, and *AD1_TPS104*), two private *TPSs* (*AD1_TPS100* and *AD2_TPS125*), and one atypical *TPS* (*AD2_TPS41*). Also, a TPS-associated coexpression module of eight genes involved in the terpenoid biosynthesis pathway was identified in the transcriptomic data of herbivore-infested *G. hirsutum*. These findings will help us understand the contributions of *TPS* family members to interspecific terpene chemotypes within *Gossypium* and offer valuable resources for breeding insect-resistant cotton cultivars.

## 1. Introduction

To withstand the negative effects of a broad array of biotic stresses, including attack from diverse herbivores, plants have developed highly sophisticated strategies [1,2]. In particular, in response to insect herbivory, plants emit a specific blend of herbivore-induced plant volatiles (HIPVs) [3,4]. HIPVs mainly comprise fatty acid derivatives (i.e., green leaf volatiles), phenylpropanoids/benzenoids, and terpenoids. Among these, terpenoids represent the largest and most structurally diverse group, mainly encompassing C_10_ monoterpenes, C_15_ sesquiterpenes, C_11_ and C_16_ homoterpenes, and some C_20_ diterpenes [5]. In plants, volatile terpenes serve multiple ecological functions in their interactions with the surrounding organisms [6]. One relatively well-studied ecological function of herbivore-induced terpene volatiles is the attraction of natural enemies of the feeding herbivores (terpene-mediated indirect defenses) (reviewed in Refs. [3,5,7,8]). Various terpenes serve as direct repellents or to exhibit direct detrimental effects on herbivores, functioning as terpene-mediated direct defenses [1]. Some herbivore-induced terpenes can function as aerial priming elicitors to trigger a defense response in uninfested tissues, enabling plants to respond faster and more strongly to subsequent herbivore attacks (terpene-mediated defense priming) (reviewed in Refs. [9,10,11]).

In plants, volatile terpenes are biosynthesized from the C_5_ isoprene precursors isopentenyl diphosphate (IPP) and dimethylallyl diphosphate (DMAPP) through either the plastidic 2-*C*-methyl-d-erythritol 4-phosphate (MEP) pathway or the cytosolic mevalonate (MVA) pathway. Generally, monoterpenes (C_10_) and diterpenes (C_20_) are typically synthesized by plastid-localized terpene synthases (TPSs, specifically, mono- and diterpene synthases), which utilize geranyl diphosphate (GPP, C_10_) and geranylgeranyl diphosphate (GGPP, C_20_) as substrates, respectively. In contrast, sesquiterpenes (C_15_) are produced by sesquiterpene synthases in the cytosol using farnesyl diphosphate (FPP, C_15_) as the substrate [12]. However, over the last decade, cross-talk between the plastidic MEP pathway and the cytosolic MVA pathway has also been reported [13]. In most plants, the *TPS* genes are the key genes in the biosynthesis of volatile terpenes and constitute a mid-size family with approximately 20 to 150 genes [14]. Based on the phylogenetic analysis of full-length amino acid sequences, the plant TPS family includes eight subfamilies: TPS-a, TPS-b, TPS-c, TPS-d, TPS-e, TPS-f, TPS-g, and TPS-h [14]. TPS-d is specific to gymnosperms, TPS-h is specific to *Selaginella* spp., and TPS-a, TPS-b, and TPS-g are exclusive to angiosperms. In angiosperms, members of the TPS-c and TPS-e subfamilies primarily function in primary metabolism, while the synthesis of terpene volatiles (e.g., secondary metabolites) are mainly attributed to members of the TPS-a, TPS-b, TPS-g, and TPS-f subfamilies. Generally, the TPS-a subfamily members catalyze the formation of sesquiterpenes, while the TPS-b and TPS-g subfamilies primarily contain monoterpene synthases, which produce cyclic and acyclic monoterpenes, respectively. TPS-f contains some diterpene synthases, which contribute to the formation of C_11_ and C_16_ homoterpenes [1,14,15].

The increasing availability of plant genome resources has facilitated genome-wide and systematic analysis of the *TPS* gene family. For example, based on a single reference genome, members of the *TPS* gene family have been systematically identified in multiple angiosperm species, such as *Actinidia chinensis* [16], *Arabidopsis thaliana* [17], *Artemisia argyi* [18], *Brassica rapa* [19], *Cannabis sativa* [20], *Cinnamomum camphora* [21], *Citrus sinensis* [22], *Dendrobium chrysotoxum* [23], *Eucalyptus grandis* and *E. globulus* [24], *Lathyrus odoratus* [25], *Malus domestica* [26], *Phoebe bournei* [27], *Pogostemon cablin* [28], *Setaria italica* [29], *Solanum lycopersicum* [12,30], and *Vitis vinifera* [31]. Recently, there has been a rapid increase in the number of plant pangenome studies, which encompass many genomes and can provide deeper insights into the overall genetic diversity and the natural phenotypic variation compared to analyses based on one reference genome [32]. Moreover, the third-generation sequencing technologies, which offer long reads and highly efficient detection of structural variations (SVs), have started to replace previous next-generation sequencing (NGS) technologies in the construction of pangenomes for plant species such as soybean [33], tomato [34], rice [35], and cotton [36]. These pangenome data have laid the foundation for genome-wide identification and deeper analysis of gene families and functional studies. For instance, Sun and coworkers [37] identified 45 *TPS* genes (comprising 32 core and 13 variable genes) in rice using a high-quality pangenome of 33 accessions reported by Qin et al. [35]. They [38] subsequently identified thirty-one *TPS* genes (including twenty core genes, three near-core genes, seven dispensable genes, and one private gene) in maize based on a pangenome derived from twenty-six high-quality genomes constructed by Hufford et al. [39].

Cotton (*Gossypium* spp.), especially the two cultivated allotetraploid species *G. hirsutum* (AD)1 and *G. barbadense* (AD)2, is grown worldwide for its fiber and seed oil, but the plants are attacked by numerous herbivorous insect species, such as *Helicoverpa armigera* and *Apolygus lucorum*. In response, these plants have evolved sophisticated defense mechanisms that include a wide range of compounds, including cadinene-type sesquiterpene aldehydes (e.g., gossypol and related derivatives) and a complex blend of mono- and sesquiterpenes (the HIPVs discussed above). The monoterpenes α-pinene, β-pinene, β-myrcene, γ-terpinene, linalool, limonene, α-thujene, α-terpinene, terpinolene, and p-cymene; the sesquiterpenes β-caryophyllene, α-humulene, β-farnesene, δ-guaiene, and δ-cadinene; and the homoterpenes 4,8-dimethylnona-1,3,7-triene (DMNT) and 4,8,12-trimethyltrideca-1,3,7,11-tetraene (TMTT) have been identified in either cultivated or wild cotton plants [1,40,41,42,43]. Among these compounds, several constitutive volatile terpenes are stored in pigment glands (e.g., α-pinene, β-pinene, myrcene, and caryophyllene) and are immediately released upon mechanical damage and herbivore attack. Other terpenes, such as (*E*)-β-ocimene, linalool, DMNT, and (*E*)-β-farnesene, are synthesized de novo and released more than 24 h after herbivore infestation and are induced only by herbivore attack [40]. Numerous diverse *TPS* genes are responsible for this terpene diversity in the cotton genome; members of the *TPS* gene family have been identified in *G. hirsutum* (AD_1_), *G. barbadense* (AD_2_), *G. arboreum* (A_2_), and *G. raimondii* (D_5_) based on their respective genomes. For instance, Cui et al. [44] identified 71, 75, 60, and 54 *TPS* genes in these respective species, whereas Mehari et al. [45] reported 84, 86, 70, and 64 *TPS* genes.

The extensive genomic diversity of *Gossypium*, with eight diploid genome types (A–G, K) and one allopolyploid clade (AD-genome), has long attracted considerable attention [36,46]. The genomes of over 20 *Gossypium* species, including diploid and allotetraploid species, have been sequenced to date [36,46]. What is more, pangenomes of the *Gossypium* genus are becoming increasingly attractive in recent years and have been constructed in some studies [36,47,48], opening up new avenues to compare the homology and diversity of the *TPS* family genes across cotton species, and thereby more accurately revealing the evolution and function of *TPS* gene families.

We previously identified volatile terpenes emitted from *G. hirsutum* and *G. barbadense* plants when infested by *H. armigera* and *A. lucorum* and characterized the function of fifteen TPS proteins in *G. hirsutum* and one TPS in *G. barbadense*, which contribute to the accumulation of several terpene components [1,49,50,51,52,53]. To comprehensively investigate variations in, and potential functions of, the *TPS* gene family across various cotton species, here, we first constructed a gene-based pangenome of the *Gossypium* genus in this study by integrating the genomes of twenty diploid and seven tetraploid species. Members of the *TPS* gene family in this pangenome were then identified based on the genomic coordinates of gene models and sequence similarity. Gene structure analysis of representative *TPS* genes across the population were also analyzed. We also used transcriptomic data from cotton plants infected by insects to analyze the expression profiles of *TPS* genes and to identify TPS-associated coexpression modules. The gene-based pangenome of the *Gossypium* genus constructed here will provide a rich resource for future functional studies on allelic TPS variants across various cotton species.

## 2. Results

### 2.1. A Syntelog-Based Pangenome of Gossypium Genus

**Figure 1:**
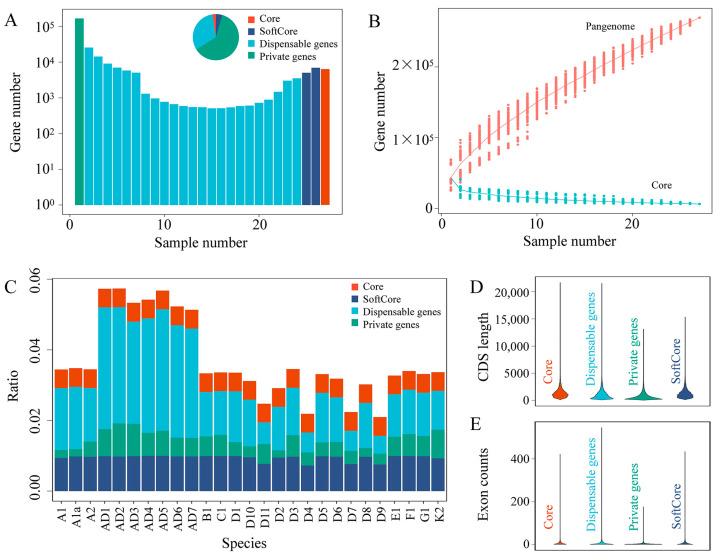
Overview of syntelog-based *Gossypium* pangenome. (**A**) Composition of gene types in the pangenome. (**B**) Variation in number of pangenes (red) and core genes (blue) with increasing number of sampled genomes. (**C**) Ratios of core, softcore, dispensable, and cloud genes across 27 *Gossypium* species genomes, with the total count of pangenes normalized to 1. (**D**) CDS length distribution of core, softcore, dispensable, and private genes in the pangenome. (**E**) Exon counts of core, softcore, dispensable, and private genes in the pangenome. Core genes are present in all 27 genomes; softcore genes are present in over 90% of the 27 genomes; dispensable genes are present in more than 1 but less than 90% of the 27 genomes; and private genes are exclusive to a single genome.

A total of 27 representative genomes (Table S1) for the 20 accessions from 19 diploid *Gossypium* species, comprising 3 A-genome accessions (A1a, A1, and A2), 11 D-genome species (D1–D11), 6 additional diploid *Gossypium* species (B1, C1, E1, F1, G1, and K2 genomes), and 7 tetraploid *Gossypium* species (AD1–AD7), were selected to construct the pangenome for *Gossypium* using the synteny-based gene family clusterer tool SynPan (https://github.com/dongyawu/PangenomeEvolution, accessed on 16 October 2023) [54]. The genes from these 27 genomes were classified into 269,549 syntelog groups (SGs), including 6371 core, 11,994 softcore, 84,866 dispensable, and 166,318 private SGs (Figure 1A). In the pangenome analysis, the total number of genes continued to increase with the addition of genomes, while the number of core genes gradually decreased and tended to be relatively stable (Figure 1B). Among the 27 *Gossypium* genomes, the 7 tetraploid genomes, as expected, contained more genes than their diploid counterparts, with the AD5 genome having the most genes (78,303) and the diploid D9 genome having the least (26,030). Dispensable genes constituted the largest proportion of the genes in all analyzed genomes; the presence of core and softcore genes remained relatively consistent, and private genes were the most variable across the 27 genomes (Figure 1C). Furthermore, the length distribution of coding sequences (CDSs) and the exon numbers for core, softcore, dispensable, and private genes were assessed, which revealed that the average CDS was longest for core genes, followed by dispensable genes, softcore genes, and private genes (Figure 1D). Dispensable genes had the most exons, followed by softcore and core genes, with private genes having the fewest (Figure 1E).

### 2.2. Identification of TPS Gene Family Based on Gossypium Pangenome

In the pangenome of *Gossypium*, 208 TPS SGs were identified (Table S2), including 2 core, 6 softcore, 131 dispensable, and 69 private SGs. Notably, two core SGs (TPS5 and TPS42) and six softcore SGs (TPS11, TPS12, TPS13, TPS35, TPS37, and TPS47) were present in 25 to 27 of the analyzed genomes, indicating that these genes are relatively stable during the domestication process.

**Figure 2:**
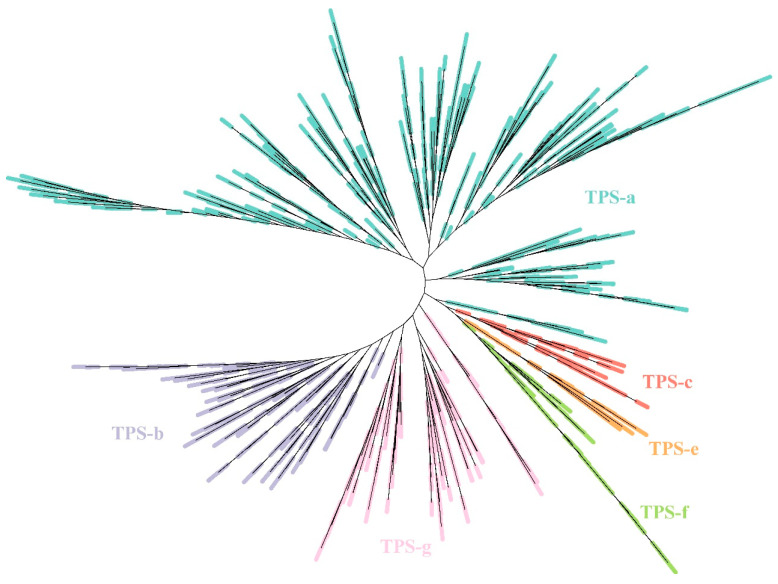
Phylogenetic tree of terpene synthases encoded by genes from *Arabidopsis* and *Gossypium* pangenome.

The phylogenetic tree based on the amino acid sequences of TPSs from the *Arabidopsis* and *Gossypium* pangenome revealed that cotton TPSs clustered into six recognized angiosperm TPS subfamilies (TPS-a, TPS-b, TPS-c, TPS-e, TPS-f, and TPS-g), excluding TPS-d (specific to gymnosperm) and TPS-h (specific to *Selaginella* spp.) (Figure 2). The TPS-a subfamily, which contributes to the formation of sesquiterpene, contained the highest number of TPSs (190 cotton TPSs), representing over half of the phylogenetic clades, followed by the TPS-b subfamily (51 TPSs), TPS-c (4 TPSs), TPS-e (6 TPSs), TPS-f (15 TPSs), and TPS-g (96 TPSs).

**Figure 3:**
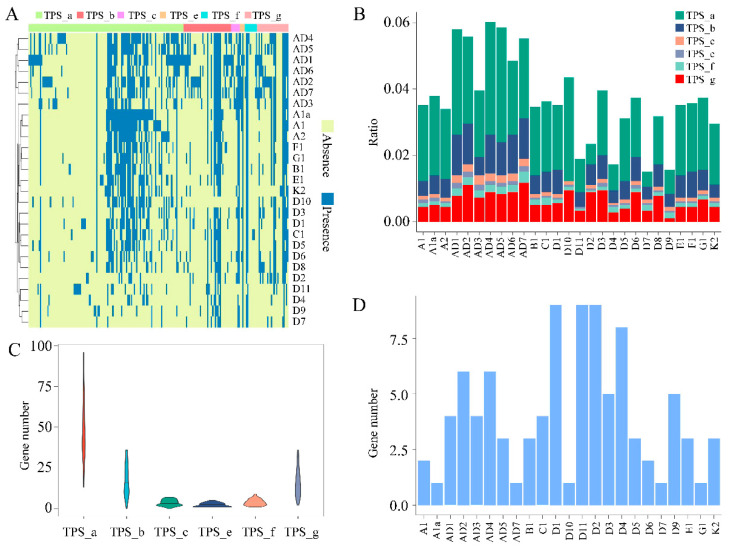
*TPS* gene family in the *Gossypium* pangenome. (**A**) Heatmap of presence/absence variation (PAV) for 206 variable *TPS* genes across six subfamilies (TPS-a–c, TPS-e–g) in 27 *Gossypium* species genomes. (**B**) Ratios of identified genes for each of the six subfamilies across 27 *Gossypium* species genomes. (**C**) Number of identified genes in each of the six subfamilies in the *Gossypium* pangenome. (**D**) Number of *TPS* genes exclusive to a single genome.

Presence/absence variation (PAV) analysis of variable *TPS* genes, including softcore, dispensable, and private genes, across six subfamilies (TPS a–c, TPS e–g) in the 27 *Gossypium* genomes revealed that the D7-, D9-, D4-, and D11 genomes had substantial losses in *TPS* genes, retaining only 21, 25, 26, and 31 genes of the 208 identified *TPS* genes, respectively (Figure 3A). In terms of the number of *TPS* genes across all the *Gossypium* genomes, AD1 contained the most *TPS* genes (92), not including tandem duplications, while AD4 had the most (108) when tandem duplication events were included (Figure 3B). For the TPS subfamilies, the D11 genome lacked the TPS-c subfamily genes (Figure 3B). The TPS-a subfamily had the most genes, while the TPS-c, TPS-e, and TPS-f subfamilies contained the fewest (Figure 3C). Sixty-nine private *TPS* genes, exclusive to single genomes, were distributed across 23 species, with the exception of AD6, D8, F1, and A2 (Figure 3D). Notably, despite its lower total of *TPS* genes (31), the D11 genome had the most private TPS genes (9) among all studied species. The gene features of these *TPS* private genes, such as the CDS length, gene length, exon number, and Pfam domain number, were comparable to those of other TPS genes (Figure S1).

### 2.3. Mutational Load Analysis and Selection Pressure Analysis of Identified TPS Genes

**Figure 4:**
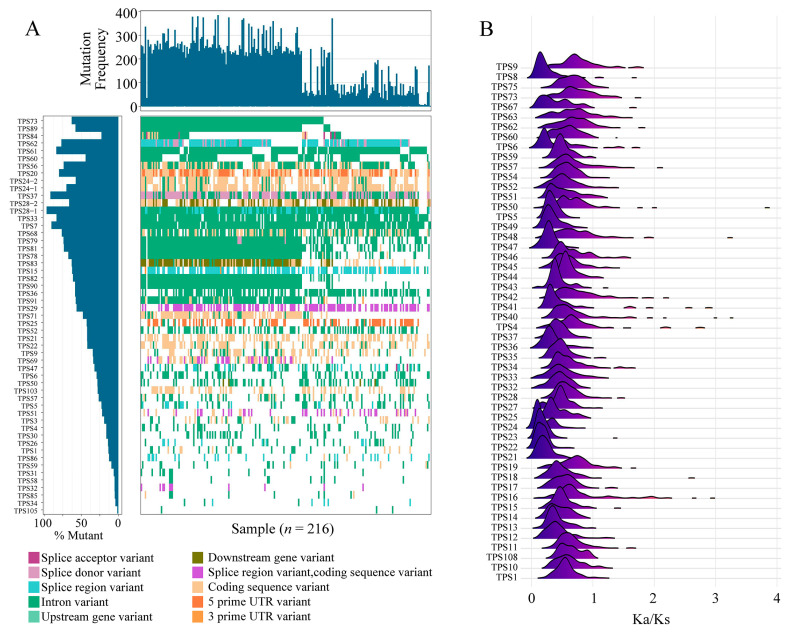
Selection pressure analysis. (**A**) Waterfall plot of the variation burden of *TPS* genes in the 216 *Gossypium* accessions. (**B**) The distribution of Ka/Ks for each *TPS* gene in 27 samples.

Among the 216 accessions, 49 *TPS* genes mainly had 10 types of variants: the splice acceptor variant, splice donor variant, splice region variant, splice region variant, intron variant, upstream/downstream gene variant, coding sequence variant, and 5′/3′ UTR variant. In particular, numerous splicing variants, such as the splice acceptor variant, splice donor variant, splice region variant, and intron variant occurred in these *TPS* genes (Figure 4A). The gene *TPS19-2* had the most variability, predominantly featuring intron and splice region variants, while its homolog *TPS19-1* had less mutation rate variability and different types of mutations, primarily coding sequence and downstream gene variants.

The nonsynonymous/synonymous (Ka/Ks) value for each *TPS* gene was calculated, and genes with Ka/Ks values < 4 were visualized (Figure 4B). The results revealed that these 52 *TPS* values peaked between 0 and 1, with those of *TPS8*, *TPS21*, *TPS22*, *TPS23*, and *TPS24* close to 0, indicating that these genes are highly conserved and subject to strong purifying selection.

### 2.4. Gene Structure Analysis of Representative TPS Genes across Cotton Species

**Figure 5:**
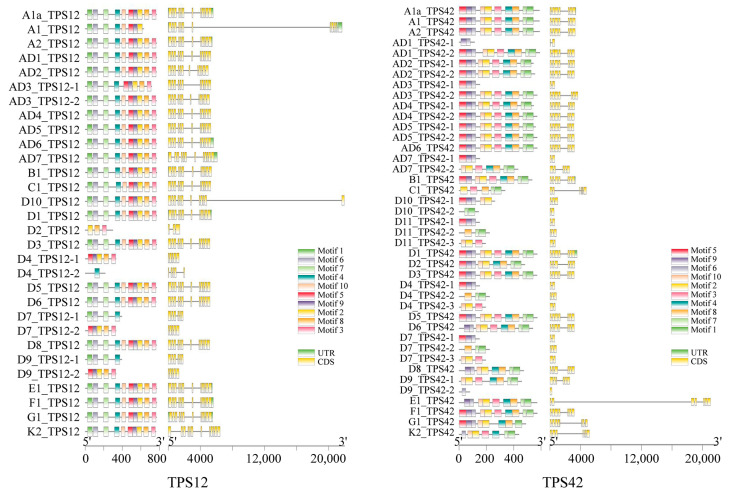
Structure of representative *TPS* genes *TPS12* and *TPS4* across various *Gossypium* species.

The gene structures of paralogous *TPS* genes located in collinear regions across various cotton genomes were analyzed, and six representative *TPS* genes, one from each of the subfamilies (TPS-a–TPS-c and TPS-e–TPS-g), were selected for structural visualization (Figure 5 and Figures S2–S7). Some genes, such as *D_4__TPS12-1*, *D_7__TPS12-2*, *D_9__TPS12-2*, *AD_7__TPS42-2*, and *D_11__TPS42-2*, lacked motifs at the beginning of their sequences, while others, such as *D_7__TPS12-1*, *D_9__TPS12-1*, *D_11__TPS42-1*, and *D_4__TPS42-1*, were missing motifs at the end. A few genes, including *D_4__TPS12-2* and *D_9__TPS42-2*, had lost motifs at both the beginning and the end, retaining only a central motif (Figure 5).

### 2.5. Atypical TPS Genes in the Gossypium Pangenome

**Figure 6:**
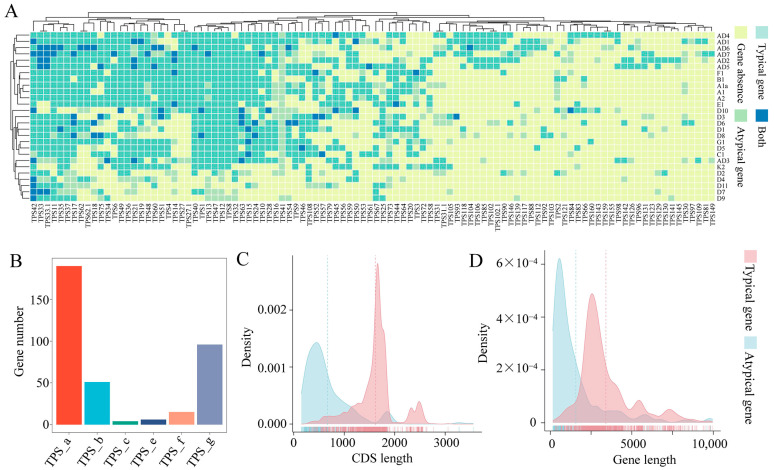
Atypical *TPS* genes in *Gossypium* pangenome. (**A**) Heatmap for atypical *TPS* genes across 27 *Gossypium* species genomes. “Both” indicates the presence of both typical and atypical *TPS* genes in the same species. (**B**) Count of atypical *TPS* genes in each of the six subfamilies (TPS-a–c, TPS-e–g). (**C**) CDS length distribution and (**D**) gene length distribution of atypical and typical *TPS* genes.

TPS family members that lacked conserved domains were considered atypical genes; typical genes retained the conserved domains. In this study, we identified 362 atypical genes derived from 102 individual *TPS* SGs. For example, *TPS41* was identified as an atypical gene in 23 cotton species, as a typical gene only in AD1, and as absent in D4, D7, and D11 (Figure 6A). Forty TPS genes had both typical and atypical counterparts in certain species. For instance, in D10, TPS84 had one typical gene (*D10_TPS84-1*) and one atypical (*D10_TPS84-2*), which could be largely due to segmental duplications and subsequent losses. The TPS-a subfamily contained the most atypical genes, followed by the TPS-g subfamily, and the TPS-e had the fewest atypical genes (Figure 6B). When the CDS length and overall gene length between atypical and typical *TPS* genes were compared, atypical *TPS* genes generally had shorter CDSs and overall gene lengths than typical genes did (Figure 6C,D).

### 2.6. Identification of Herbivory-Responsive TPS Genes Using RNA-Seq Data

**Figure 7:**
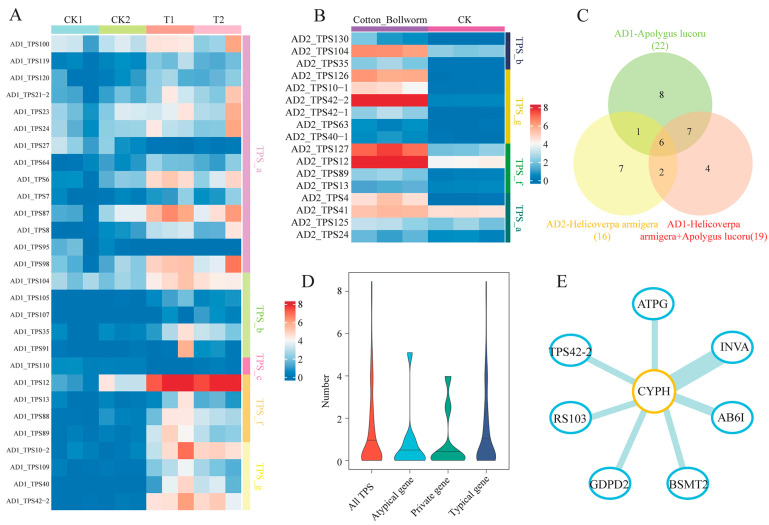
Herbivory-responsive *TPS* genes. (**A**) Heatmaps of the expression patterns of *TPS* genes in AD1-genome species infested with herbivores. T1, plants of *Gossypium hirsutum* (cotton) infested with *Apolygus lucorum* alone (CK1, control plants); T2, plants simultaneously infested with *A. lucorum* and *Helicoverpa armigera* (CK2, control plants). (**B**) Heatmaps of the expression patterns of *TPS* genes in AD2-genome species infested by *H. armigera* alone. (**C**) Venn diagram of common and unique TPSs under different treatments. (**D**) Distribution of expression levels for all *TPS* genes, atypical, private, and typical *TPS* genes. (**E**) A coexpression module involved in the TPS pathway. AB6I, ABC transporter I family member 6; ATPG, ATP synthase gamma chain; BSMT2, benzoic acid/salicylic acid carboxyl methyltransferase 2; CYPH, peptidyl-prolyl cis-trans isomerase; GDPD2, Glycerophosphodiester phosphodiesterase 2; INVA, scid beta-fructofuranosidase; RS103, small ribosomal subunit protein eS10x.

The expression profiles of *TPS* genes were analyzed using the RNA-seq data of cotton leaves infested for 36 h with *H. armigera* alone, *A. lucorum* alone, or both pests. In the AD_1_ genome, 19 of 22 herbivory-responsive *TPS* genes were upregulated in cotton leaves infested with *A. lucorum* alone, while almost all (18 of 19) differentially expressed *TPS* genes were upregulated when infested with both pests (Figure 7A). In the AD_2_ genome, all 17 *TPS* genes were induced after *H. armige*ra infestation (Figure 7B). Interestingly, of these 17 *H. armigera*-induced *TPSs*, *AD_2__TPS41* was atypical, which might be overlooked in traditional gene family identification and analyses based on a single genome. The Venn diagram indicates that six *TPS* genes (TPS10, TPS12, TPS40, TPS42, TPS89, and TPS104) were differentially upregulated in all three treatments (Figure 7C). These six TPSs were previously characterized in *G. hirsutum* (AD_1_) [1]. Notably, two private genes (*AD_1__TPS100* and *AD_2__TPS125*) were also upregulated. The expression levels of most private, atypical, and typical TPS genes were low (Figure 7D).

In many specialized metabolic pathways, functionally associated genes often have similar expression patterns and form tight modules within coexpression networks. Therefore, a coexpression analysis is valuable for screening transcriptome data for functionally related genes [55]. We thus retrieved and analyzed transcriptomic data from cotton plants after herbivore infestation to identify coexpressed modules within the terpene biosynthesis pathway using the gene coexpression network-based approach developed by Wisecaver et al. [55]. In the transcriptomic data of the AD_1_ genome, a TPS-associated coexpression module comprising eight genes involved in the terpenoid biosynthesis pathway was identified, with *peptidyl-prolyl cis-trans isomerase* as a central player (Figure 7E), suggesting that it has a pivotal role.

## 3. Discussion

In the gene-based pangenome of the *Gossypium* genus that we constructed by integrating the genomes of twenty diploid and seven tetraploid species, 269,549 syntelog groups (SGs) were identified (Figure 1 and Table S1), far exceeding the 36,496 SGs reported for the diploid *Gossypium* pangenome by Wang et al. [36]. These extensive data were then used to comprehensively investigate *TPS* family members across the various *Gossypium* species. Angiosperm species harbor various numbers of *TPS* genes, ranging from 40 in *Arabidopsis* to 152 in grapevine (*Vitis vinifera*) [14]. Traditionally, genome-wide identification of the TPS family members has primarily been based on a single genome. For example, previous studies identified the TPS family members in *G. hirsutum* (AD_1_), *G. barbadense* (AD_2_), *G. arboreum* (A_2_), and *G. raimondii* (D_5_) using their respective genomes [1,44,45,56,57,58]. Cui et al. [44] reported the presence of 71, 75, 60, and 54 *TPS*s in these respective species, while Mehari et al. [45] identified 84, 86, 70, and 64 *TPS*s. Additionally, Liu et al. [56] identified 110, 115, and 69 *TPS* genes in the *G. hirsutum*, *G. barbadense*, and *G. raimondii* genomes, respectively. Also in the *G. hirsutum* genome, Wen et al. [57] identified 76 *TPS* genes, in contrast to the 107 reported by Liu et al. [59]. Here, we identified 208 *TPS* genes across 27 cotton species in this pangenome, including 105 in *G. hirsutum* (AD_1_), 100 in *G. barbadense* (AD_2_), 61 in *G. arboreum* (A_2_), and 56 in *G. raimondii* (D_5_) (Table S2). Possible explanations for the observed variation in the number of family members across these cotton species between previous studies and our study could, firstly, be attributed to different genome assembly versions. In addition, the synteny-based gene family clusterer SynPan, used for homologous gene screening in the present study, is relatively stringent. Also, in traditional gene family analyses based on single genome references, atypical TPSs that lack both the C-terminal and N-terminal conserved domains were overlooked. In fact, here, we found 16, 11, 6, and 4 atypical genes in *G. hirsutum* (AD_1_), *G. barbadense* (AD_2_), *G. arboreum* (A_2_), and *G. raimondii* (D_5_), respectively (Figure 6 and Table S2).

In our pangenome of *Gossypium*, the TPS-a subfamily contained the most TPSs (190 sesqui-TPSs), representing over half of the 362 TPS family members analyzed in the phylogenetic tree (Figure 2). The cotton TPS-a subfamily harbors numerous genes encoding cadinene synthases (CDNs), in addition to non-cadinene-type sesqui-TPSs responsible for the biosynthesis of volatile sesquiterpenes such as caryophyllene and farnesene common in various plant species. It is widely acknowledged that the enzyme CDN controls the first step in the biosynthesis of gossypol, an important defense compound uniquely produced by *Gossypium* species [57,60]. Liu et al. [56] identified 13, 19, 14, and 11 *CDN* genes (sharing > 80% nucleotide identity) in the *G. hirsutum*, *G. barbadense*, *G. arboretum*, and *G. raimondii* genomes, respectively. Wen et al. [57] discovered 21 *CDN* genes in the *G. hirsutum* genome. Here, we identified a total of 32 *CDNs* across these four species: 928 in *G. hirsutum*, 17 in *G. barbadense*, 14 in *G. arboreum*, and 11 in *G. raimondii*). This variable number of *CDN* genes across cotton species suggests recent small-scale duplication events, representing the rapid, lineage-specific evolution of crucial genes for specialized metabolites [56]. Similarly, Jiang et al. [61] revealed that plant species predominantly expand their TPS-a and TPS-b subfamilies to meet the specific needs of secondary metabolite production, reflecting adaptive responses to ecological diversification and environmental challenges.

In publications demonstrating the remarkable quantitative and qualitative plasticity of the TPS family across *Gossypium* species, various research groups have each proposed new nomenclature. This lack of a consistent nomenclature can cause significant confusion and difficulty in matching new names with the original ones without detailed comparisons of the sequences [62]. Two factors contributed to this problem. (1) Different authors selected different versions of the various genome assemblies. For instance, *G. hirsutum* alone has 19 versions (https://www.cottongen.org/organism/1033, accessed on 2 May 2024). (2) The authors overlooked the originally described names of functionally characterized cotton TPSs and suggested new and inconsistent names. To date, several *TPS* genes have been cloned and functionally characterized in cotton [1,43,49,63,64,65]. For example, the 16 previously characterized TPSs (GhTPS1–16) from *G. hirsutum* account for some of the major herbivore-induced terpene volatiles in *Gossypium* species, including (*E*)-β-ocimene, β-caryophyllene, α-humulene, α-pinene, β-pinene, β-myrcene, linalool, δ-guaiene, δ-cadinene, DMNT, and TMTT [1,43,49]. Sequence comparisons revealed that these characterized TPSs were also present in our pangenome data (Table S3). Thus, to minimize confusion about *Gossypium* TPS names, we need a consistent nomenclature that includes the existing names and is based on a generally accepted genome assembly version. Similarly, this inconsistency also occurs in the research on maize terpene synthases [62]. Köllner et al. [62] suggested standardizing maize TPS nomenclature by using “*ZmTPS*”, followed by numbers for monoterpenes and sesquiterpenes (ZmTPS1–ZmTPS36) based on the B73 reference genomes GRAMENE 4.0 and NAM 5.0 and with continued designation for copalyl diphosphate synthases (ZmTPS37/CPS1–ZmTPS41/CPS5) and kaurene synthase-like (ZmTPS42/KSL1–ZmTPS47/KSL6).

Allelic differences in *TPS* genes are crucial in both interspecific and intraspecific diversification of volatile terpenes [66,67]. For example, in the *Freesia* genus, allelic *TPS* variants account for variations in the interspecific floral volatile terpenes such as α-terpineol, β-caryophyllene, α-selinene, cadinene, and β-elemene [66]. In *Nicotiana attenuata* and maize, the intraspecific production of linalool and (*E*)-β-caryophyllene is determined by allelic variation in a linalool synthase and (*E*)-β-caryophyllene synthase, respectively [67,68]. Here, our gene-based pangenome analysis of *Gossypium* species involving 4 cultivated and 23 wild species provided insights into the interspecific variations in the cotton *TPS* family. In the pangenome of *Gossypium* genus, only 2 core and 6 softcore *TPS* genes were identified, with most *TPS* genes being categorized as dispensable (131 of 208) and private (69 of 208) (Table S2), which revealed that the interspecific variations within the *Gossypium* genus are significantly higher than the intraspecific divergence previously reported in cultivated rice (32 *Oryza sativa* accessions and 1 *O. glaberrima* accession) and maize (26 lines) [37,38]. The cultivated rice pangenome has 32 core, 13 variable, and 0 private *TPS* genes, whereas the maize pangenome has 20 core, 3 near-core, 7 dispensable, and 1 private gene. In the present study, *TPS42* was identified as a core gene (present in all species) predicted to encode an α-farnesene synthase. Its enzyme product, α-farnesene, has been reported to play important ecological roles in plant–insect interactions and plant–plant communication and plant defenses against nematodes [69]. In cotton plants, α-farnesene is an inducible compound predominantly released in response to herbivore damage rather than mechanical damage, and it is also systemically emitted from undamaged leaves exposed to priming signals [70], suggesting the ecological importance of the compound and its corresponding biosynthetic gene in the interactions of cotton plants with their environment. However, in *G. hirsutum*, AD1_TPS42 (previously known as GhTPS7) had no activity with the substrates tested in our earlier in vitro assays [1]. Similarly, AD1_TPS105 (formerly GhTPS8), AD1_TPS15-16 (formerly GhTPS9), and AD1_TPS40 (formerly GhTPS13) also seem to be nonfunctional; their function might have been lost during evolution. Previous studies demonstrated that alleles of *TPS* genes significantly diverged within closely related species, leading to species-specific gain or loss of TPS activity [25]. For example, the capacity of TPS10 variants to catalyze β-caryophyllene production varied greatly among *Freesia* species. Natural alleles from *F. speciosa*, *F. verrucosa*, *F. corymbosa*, and *F. refracta* lost this function, while FviTPS10 from *F. viridis* exhibited the highest catalytic activity and production levels [25]. Therefore, these inactive TPSs in *G. hirsutum* justify further efforts to elucidate their specific roles and the catalytic mechanisms of various allelic variants among cotton species. *TPS12* and *TPS35* were identified as softcore genes (Figure 6 and Table S2). AD1_TPS12 (formerly GhTPS14) has been shown to be responsible for DMNT biosynthesis and plays an important role in enhancing plant defense against *H. armigera* and *A. lucorum* by recruiting parasitic wasps [71,72]. A2_TPS35 from *G. arboreum* has been functionally characterized as a linalool synthase gene. Its enzyme product linalool, produced by cotton plants, is repellent to lepidopteran females and aphids [1]. The core gene *TPS5* and the softcore gene *TPS47* belong to the TPS-c gene subfamily, which encodes proteins primarily involved in primary metabolism and is not involved in volatile biosynthesis. The roles of other softcore genes, including *TPS11*, *TPS13*, and *TPS37*, encoding proteins involved in volatile biosynthesis have not yet been characterized and warrant more study.

Gene structure analysis of representative *TPS* genes across various *Gossypium* species showed that despite variations in the number of motifs within these TPS genes, the order of conserved domains in these genes remained consistent with their counterparts (Figure 5 and Figures S2–S7), suggesting that partial deletions of TPS genes resulting in a truncated protein with altered or lost functions were widespread during species diversification. In *Nicotiana attenuata*, for example, the presence/absence of a 766 bp sequence in the linalool synthase gene *NaLIS* corresponded to the variation in linalool emissions across 26 natural accessions [67]. In addition, our analysis of the transcriptomic data from infested cotton plants showed that infestations by *H. armigera* alone and *A. lucorum* alone and simultaneous infestation by both pests led to the differential expression of a total of 58 *TPS* genes, and almost all (54) were substantially upregulated (Figure 7). Among these upregulated *TPS* genes, the enzymes that were functionally characterized for *TPS4* (formerly *GbTPS1*), *TPS10* (formerly *GhTPS16*), *TPS12* (formerly *GhTPS14*), *TPS87* (formerly *GhTPS10*), *TPS89* (formerly *GhTPS15*), *TPS91* (formerly *GhTPS5*), *TPS104* (formerly *GhTPS4*), and *TPS109* (formerly *GhTPS12*) are involved in the formation of (*E*)-β-ocimene, β-myrcene, linalool, selinene, guaiene, δ-cadinene, DMNT, and TMTT. These findings are consistent with our previous studies that demonstrated increased emissions of these terpene volatiles in cotton in response to these treatments [1,51,52].

## 4. Materials and Methods

### 4.1. Construction of a Syntelog-Based Pangenome for Gossypium Using Synteny

Genomic data for the 27 cotton accessions comprising 20 diploids and 7 tetraploids obtained from CottonGen (https://www.cottongen.org/data/download, accessed on 16 October 2023) were used to construct a gene-based pangenome (Table S1), which was analyzed using SynPan (https://github.com/dongyawu/PangenomeEvolution, accessed on 16 October 2023) [54]. Genes in the pangenome were identified as core genes (present in all 27 genomes), softcore genes (present in over 90% of the 27 genomes), dispensable genes (present in more than 1 but less than 90% of the 27 genomes), or private genes (exclusive to a single genome).

### 4.2. Identification of TPS Gene Family and Construction of the Phylogenetic Tree

Hidden Markov model (HMM) profiles of the two conserved domains PF03936 (C-terminal) and PF01397 (N-terminal) for terpene synthases were downloaded from InterPro (https://www.ebi.ac.uk/interpro/, accessed on 20 October 2023) [73]. To identify the cotton TPS family in the 27 genomes, we screened protein sequences containing the two conserved domains using HMMER software (v. 3.3.2) [74] and an e-value threshold of 1 × 10^−5^. In addition, if a gene that harbored both the N-terminal and C-terminal domains was identified as a *TPS* gene in one genome, then its homologous counterparts in other genomes were also classified as belonging to the *TPS* gene family.

TPS protein sequences from *Arabidopsis thaliana* [17] were retrieved and aligned with the identified cotton TPS proteins using MAFFT software (v. 7.508) [75] and trimAl tool (v. 1.4.rev15) [76]. A phylogenetic tree was then constructed using FastTree (V. 2.1.11) [77] based on the Jones–Taylor–Thornton (JTT) model, which was visualized using the R package ggtree (http://www.bioconductor.org/packages/ggtree, accessed on 20 October 2023) [78].

### 4.3. The Mutational Load of TPS Genes in Different Gossypium Species

SNP data were obtained from the previous study [36]. Variants located within the regions of these identified cotton *TPS* genes were extracted using VCFtools (v. 0.1.16), filtered using BCFtools (v. 1.8), annotated by variant effect predictor (VEP) tool (http://www.ensembl.org/vep, accessed on 20 October 2023) [79], and visualized using R package (https://jameshoward.us/2016/02/15/waterfall-1-0-0-released/, accessed on 20 October 2023).

### 4.4. Ka/Ks Calculation

Multiple sequence alignment of the amino acids for the identified cotton TPSs was performed using MUSCLE (v. 3.8.1551) and PAL2NAL. Ka/Ks values of duplication gene pairs across different *Gossypium* species were calculated by KaKs_Calculator 2.0 with the Nei–Gojobori (NG) method [80]. Ka/Ks values < 4 were visualized using the R package ggridges (https://cran.r-project.org/web/packages/ggridges/vignettes/introduction.html, accessed on 24 October 2023).

### 4.5. RNA-Seq Analysis

RNA-seq data from cotton plants infested with insect herbivores were downloaded from NCBI (PRJNA802699 and PRJNA688359), processed using SRA-tools fastq-dump (https://github.com/ncbi/sra-tools, accessed on 24 October 2023) and fastp [81], and then mapped to the AD1 genome or AD2 genome by HISAT2 [82]. Differential expression was analyzed using DESeq2 (http://www.bioconductor.org/packages/release/bioc/html/DESeq2.html, accessed on 24 October 2023), and genes with log_2_ |Fold-change| > 1 and false discovery rate (FDR) < 0.05 were considered as differentially expressed genes (DEGs). Normalized FPKM values were calculated using Phasebook (https://github.com/phasebook/phasebook, accessed on 26 October 2023) [83] and were used to produce heatmaps using the ComplexHeatmap R package (https://github.com/jokergoo/ComplexHeatmap, accessed on 26 October 2023).

### 4.6. Conserved Motifs and Gene Structure Analysis

Conserved motifs in each TPS protein sequence across different species were predicted using the MEME Suite web server (https://meme-suite.org/meme/tools/meme; accessed on 26 October 2023), setting the number of motifs to 10. Conserved protein motifs and gene structures were visualized using TBtools (https://github.com/CJ-Chen/TBtools/releases, accessed on 26 October 2023) [84].

### 4.7. Coexpression Analysis

To discover coexpressed gene modules associated with the terpene metabolism pathway in *Gossypium*, we used coexpression network analyses as described previously [55]. The mutual rank (MR) score was calculated as described previously [85]. Then, the edge weight was calculated based on the MR using the following formulas:

$$edge\;weight1=e^{(MR-1)/5}$$

$$edge\;weight2=e^{(MR-1)/10}$$

$$edge\;weight3=e^{(MR-1)/25}$$

$$edge\;weight4=e^{(MR-1)/50}$$

$$edge\;weight5=e^{(MR-1)/100}$$

Genes with PCC > 0 and edge weight > 0.01 were processed using ClusterONE software (http://www.paccanarolab.org/cluster-one/, accessed on 26 October 2023), and modules with *p* < 0.1 were selected. The coexpression network was visualized using Cytoscape software (https://cytoscape.org/, accessed on 26 October 2023) [86].

## 5. Conclusions

Cotton (*Gossypium* spp.) plants are an economically significant crop worldwide due to their production of natural fibers that exhibit extraordinary genomic diversity and multiple distinct terpene chemotypes. The availability of extensive genomic resources and the diversity of interspecific and intraspecific chemotypes make *Gossypium* spp. an ideal model for exploring the complexity of the terpenoid metabolic network and the allelic variations in *TPS* genes. Here, we integrated genomic data from 20 diploid and seven tetraploid Gossypium species and constructed a gene-based pangenome for the genus. Our analyses of the *TPS* gene family in the pangenome led to the identification of 208 *TPS* syntelog groups (SGs), including 2 core, 6 softcore, 131 dispensable, and 69 private TPS SGs. Importantly, a total of 362 genes derived from 102 SGs were identified as atypical and truncated; these are often missed in traditional gene family identification and analyses based solely on a single genome. Our extensive data offer important insights and valuable resources for comprehensively investigating TPS family members across various cotton species and a foundation for further study to unveil the molecular mechanisms underlying the distinct terpene chemotypes in Gossypium.

## Data Availability

Data are contained within the article and Supplementary Materials.

## Supplementary Materials

The following supporting information can be downloaded at: https://www.mdpi.com/article/10.3390/ijms25179677/s1.
