# Supplementary material for: Pangenome Identification and Analysis of Terpene Synthase Gene Family Members in Gossypium
Source: Int J Mol Sci. 2024 Sep 6;25(17):9677. doi: 10.3390/ijms25179677 (PMC11395804; doi:10.3390/ijms25179677)
Supplement: Supplementary file 1 [file ijms-25-09677-s001.zip › Supplementary Materials Table S3 and Figures S1-S7.pdf]

Supplementary Materials

Pangenome Identification and Analysis of Terpene Synthase Gene Family Members in *Gossypium*

Yueqin Song [1,†], Shengjie Han [1,2,†], Mengting Wang [2], Xueqi Ni [2], Xinzheng Huang [2,*] and Yongjun Zhang [3,*]

[1] College of Horticulture and Plant Protection, Henan University of Science and Technology, Luoyang 471023, China; songyueqin6@163.com (Y.S.); hanshengjie1029@163.com (S.H.)
[2] Department of Entomology, MOA Key Lab of Pest Monitoring and Green Management, College of Plant Protection, China Agricultural University, Beijing 100193, China; wangmt@cau.edu.cn (M.W.); xueqini@163.com (X.N.)
[3] State Key Laboratory for Biology of Plant Diseases and Insect Pests, Institute of Plant Protection, Chinese Academy of Agricultural Sciences, Beijing 100193, China
* Correspondence: huangxinzheng@cau.edu.cn (X.H.); yjzhang@ippcaas.cn (Y.Z.)
† These authors have contributed equally to this work.

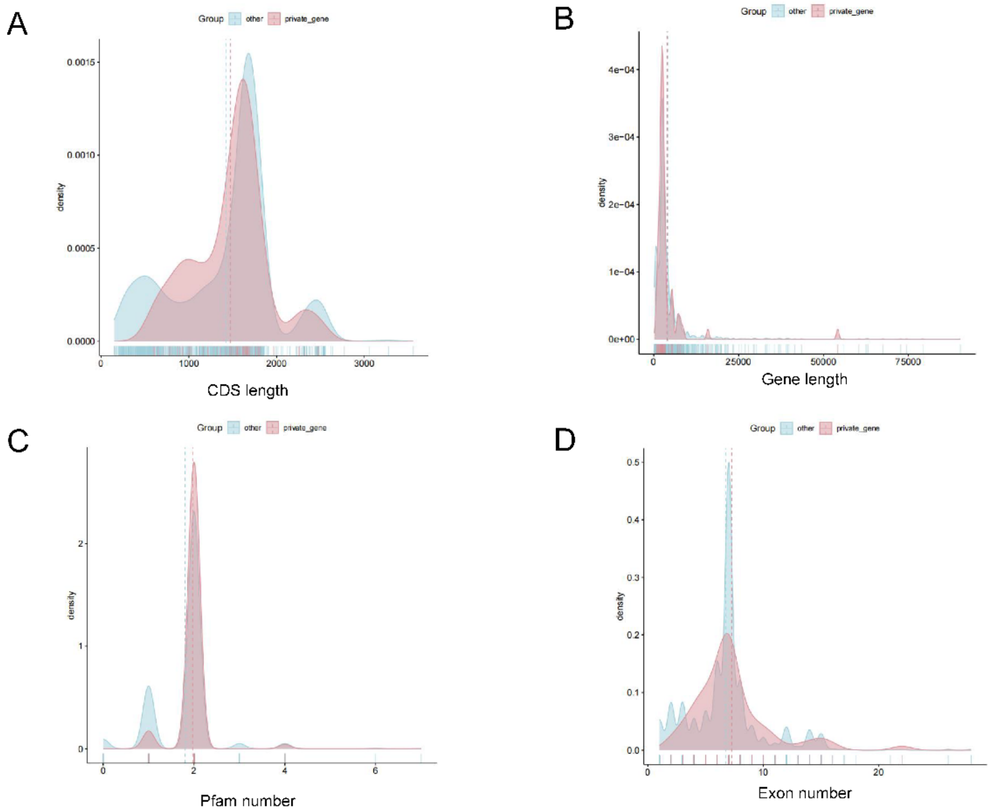

Figure S1. Comparative analysis of CDS length (**A**), gene length (**B**), pfam number (**C**) and exon number (**D**) between private genes and other genes.

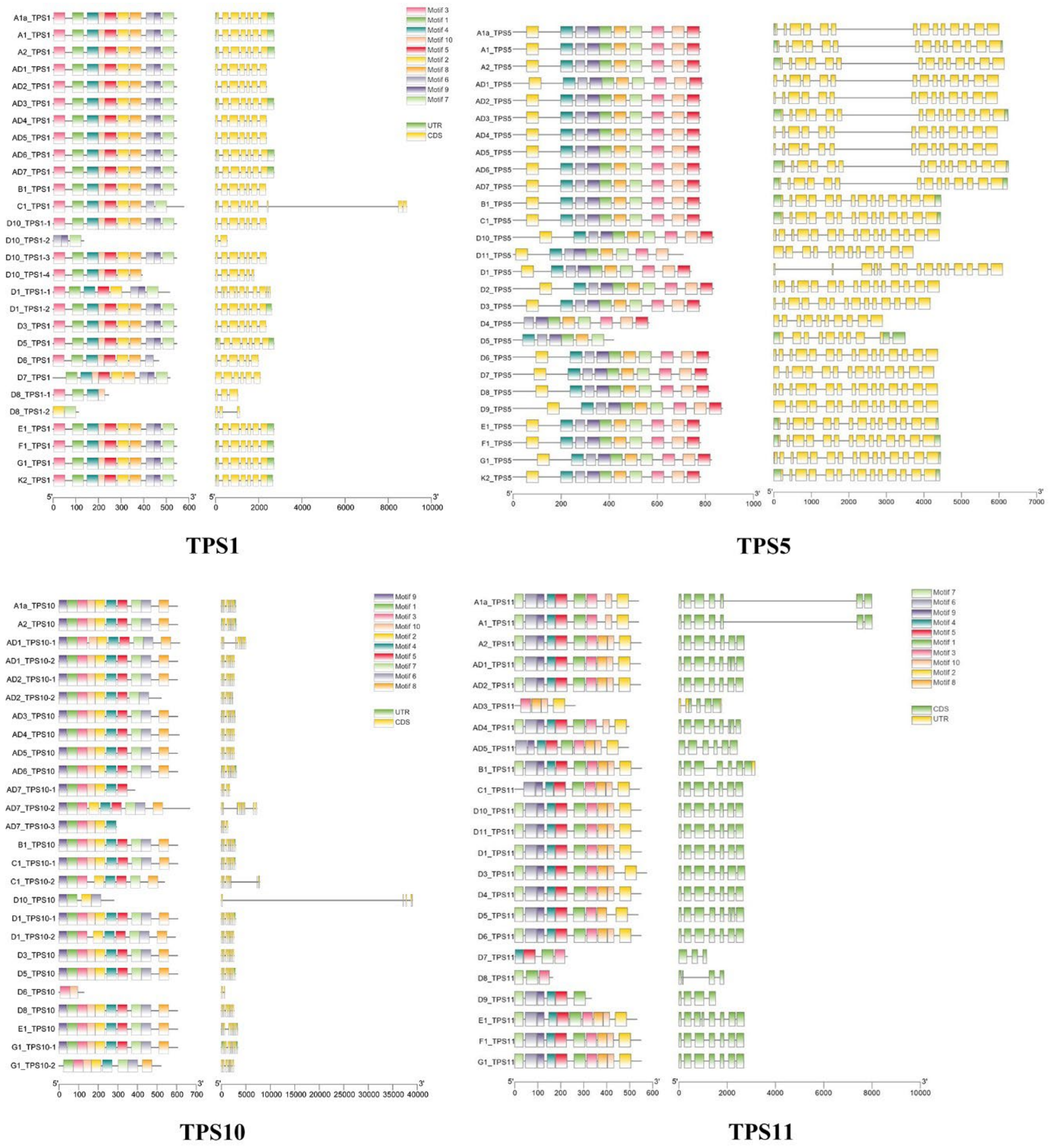

Figure S2. Structure of *TPS1, TPS5, TPS10* and *TPS11* across various *Gossypium* species.

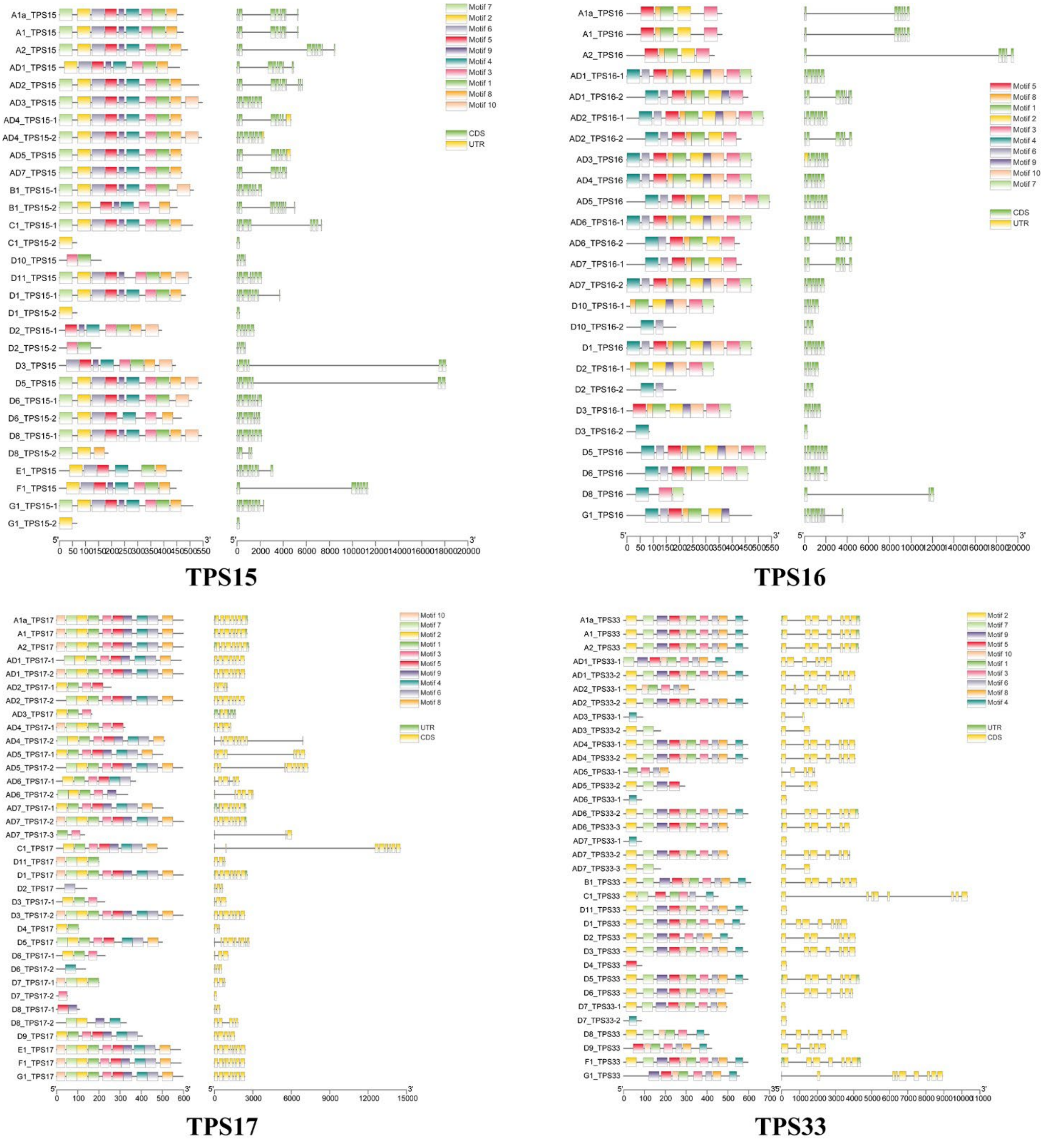

Figure S3. Structure of *TPS15, TPS16, TPS17* and *TPS33* across various *Gossypium* species.

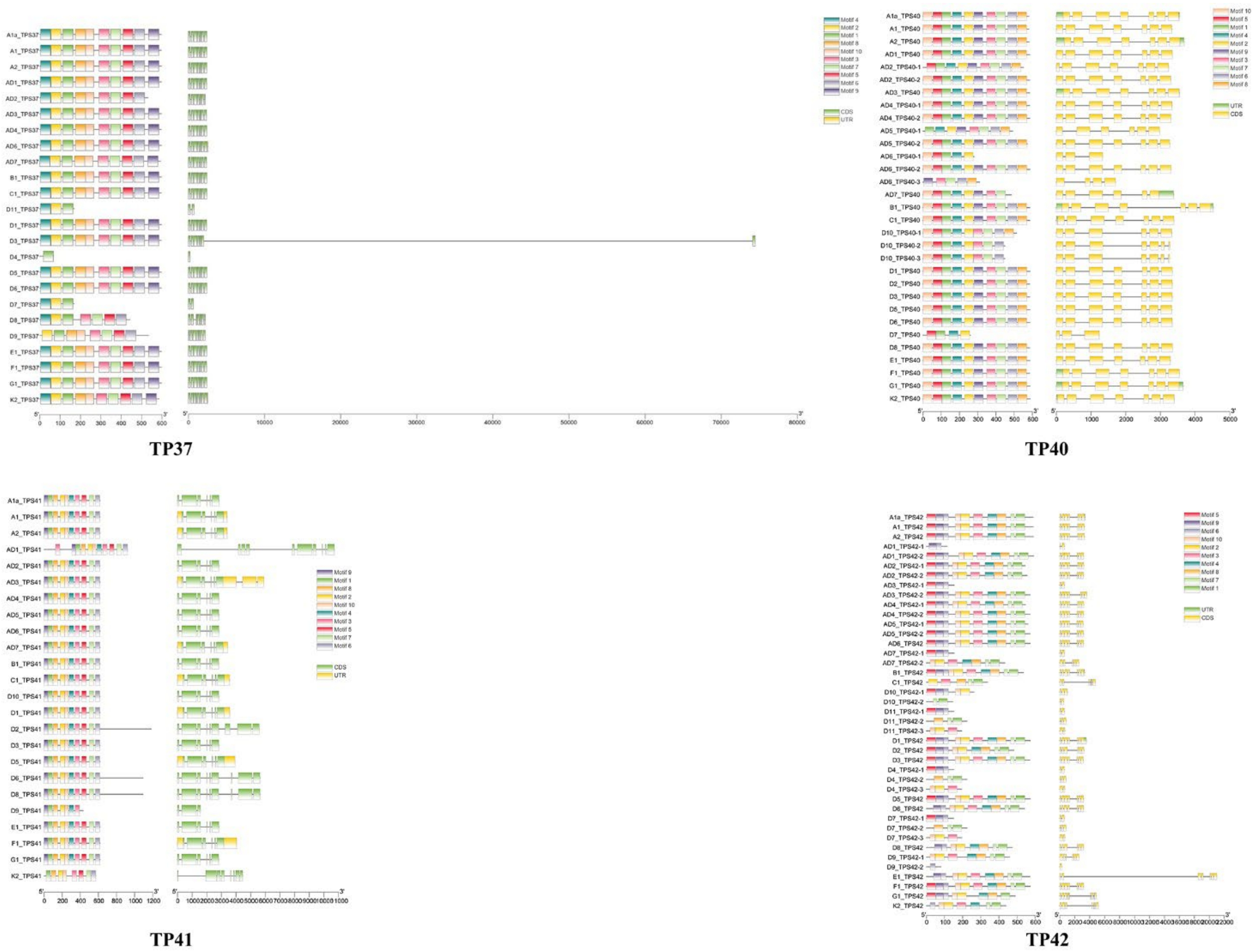

Figure S4. Structure of *TPS37*, *TPS40*, *TPS41* and *TPS42* across various *Gossypium* species.

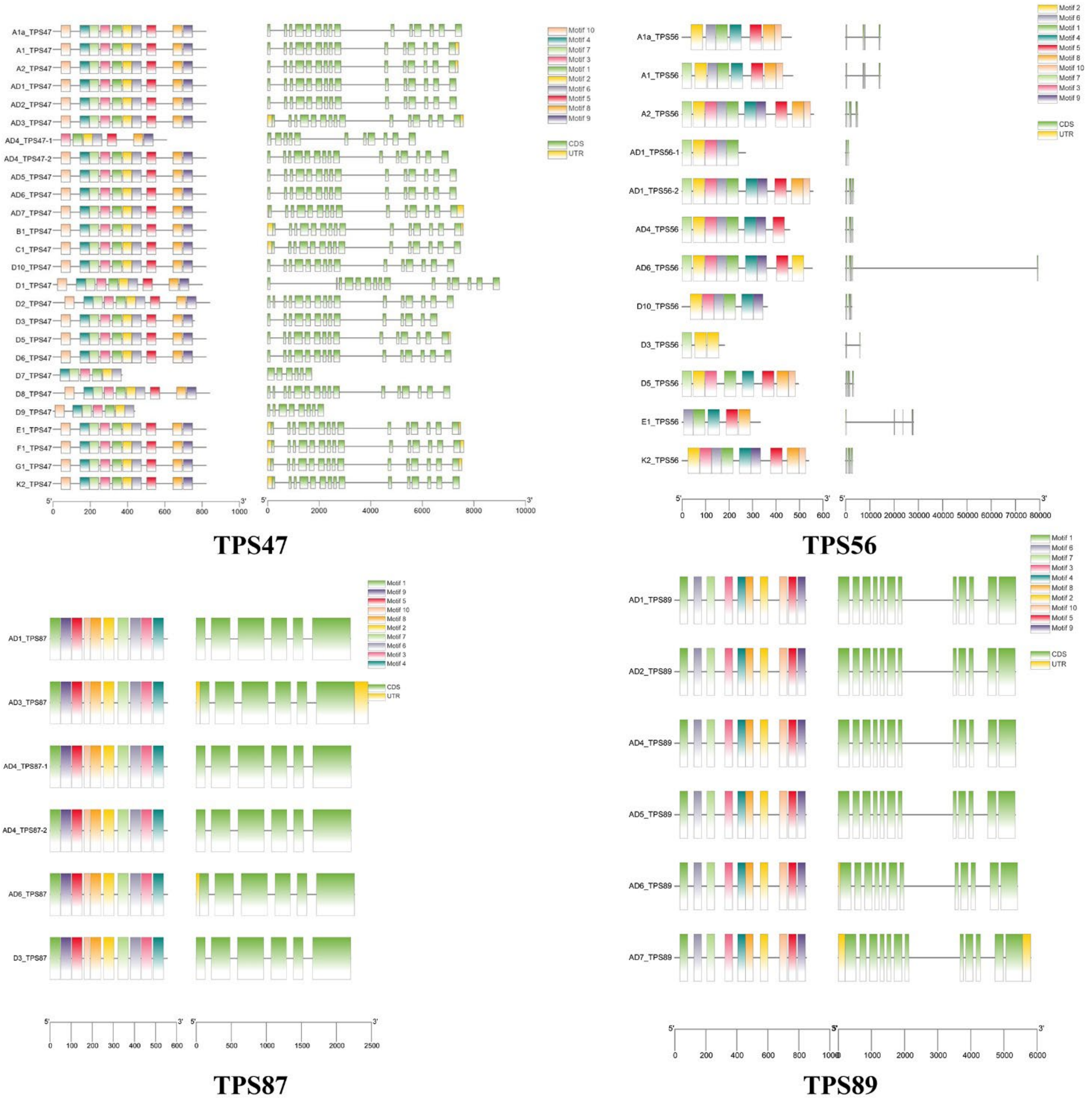

Figure S5. Structure of *TPS47, TPS56, TPS87* and *TPS89* across various *Gossypium* species.

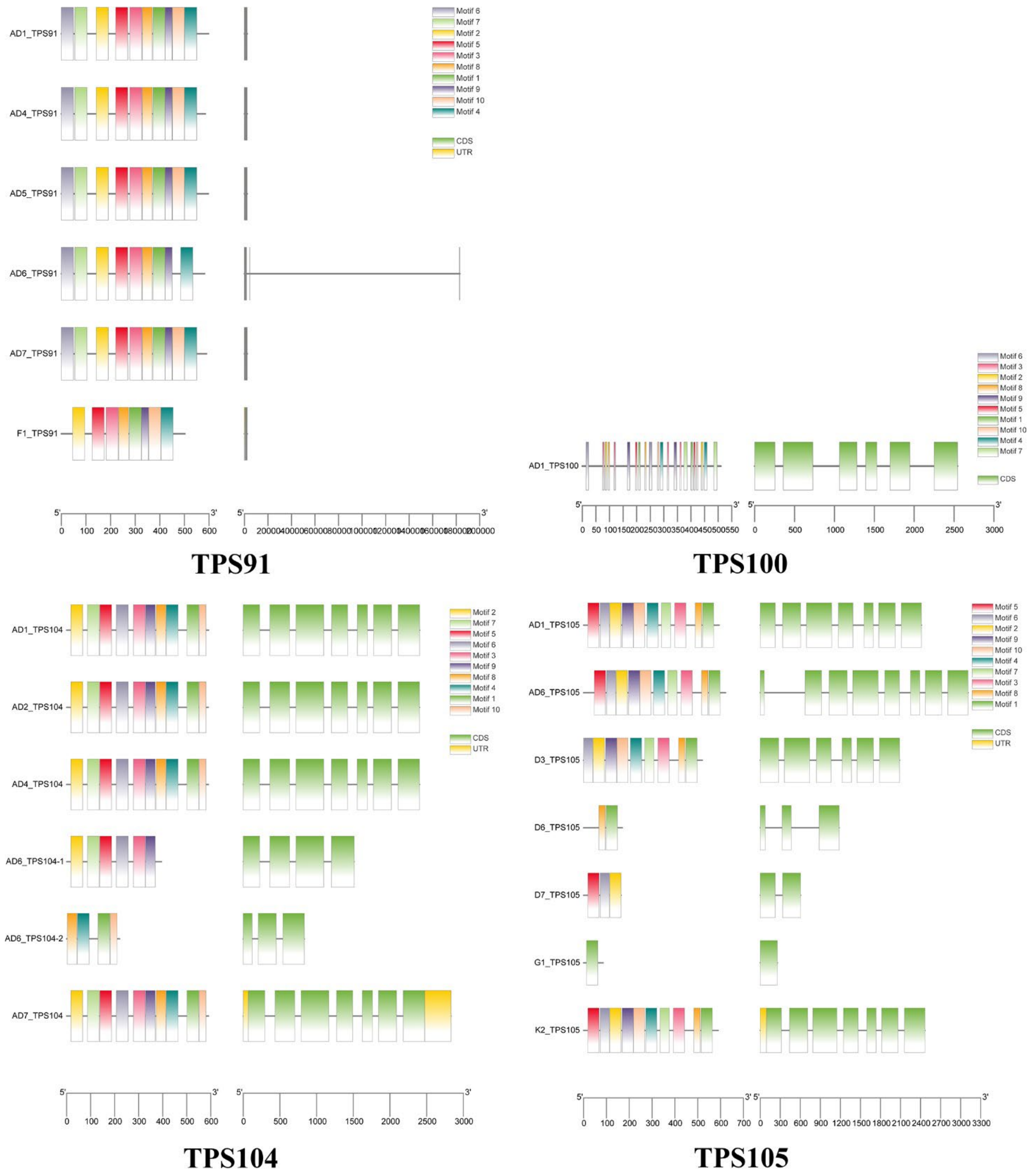

Figure S6. Structure of *TPS91, TPS100, TPS104* and *TPS105* across various *Gossypium* species.

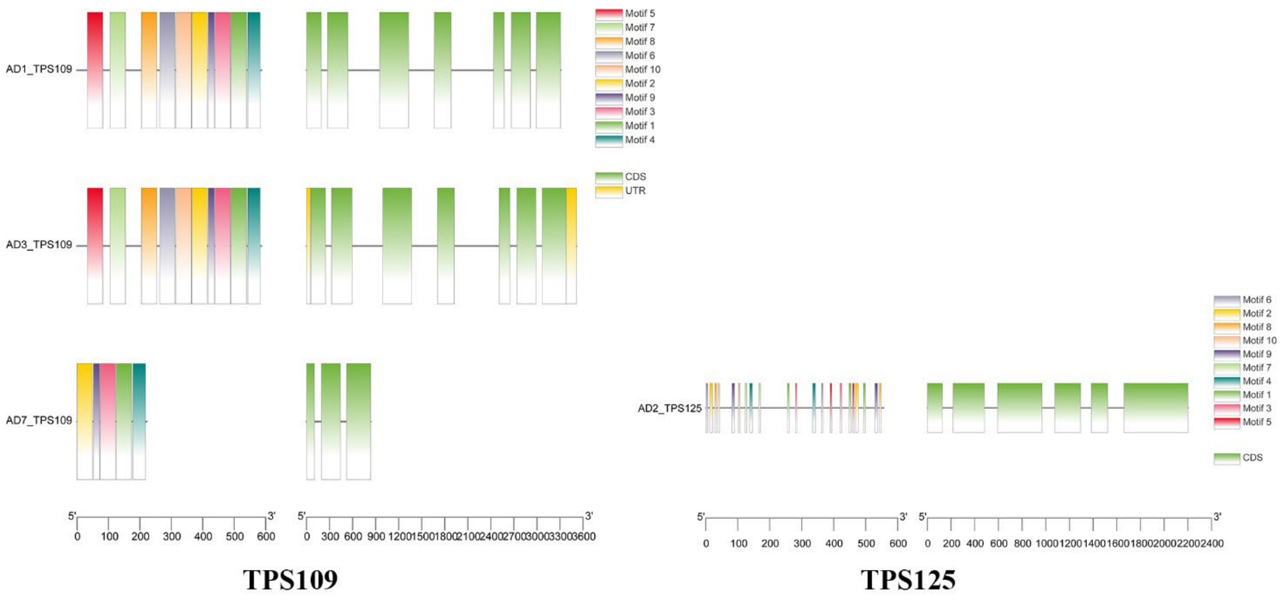

Figure S7. Structure of *TPS109* and *TPS125* across various *Gossypium* species.

Table S3. The previously functionally characterized *TPS* genes from *Gossypium hirsutum* and *G. barbadense*.

Name in This Study	Ref. [43]	Refs. [1,49,53]	Ref. [52]
TPS1	GhTPS1, KC878726	GhTPS1, JQ365627	
TPS56	GhTPS2, KC878727	GhTPS3 proposed here	
TPS33	GhTPS3, KC878728	GhTPS2, JQ957855	
TPS104		GhTPS4, KJ957820	
TPS91		GhTPS5, KJ957817	
TPS17		GhTPS6, KX963371	
TPS42		GhTPS7, KJ957816	
TPS105		GhTPS8, KX963372	
TPS15, TPS16		GhTPS9, KJ957819	
TPS87		GhTPS10, KX963373	
-		GhTPS11, KX963374	
TPS109		GhTPS12, KJ957818	
TPS40		GhTPS13, KX963375	
TPS12		GhTPS14, KX963376	
TPS89		GhTPS15, KX963377	
TPS10		GhTPS16, MT875327	
TPS4			GbTPS1, OM522661
